# Sharing Health Information Using a Blockchain

**DOI:** 10.3390/healthcare11020170

**Published:** 2023-01-05

**Authors:** Luis B. Elvas, Carlos Serrão, Joao C. Ferreira

**Affiliations:** 1 Information Sciences, Technologies and Architecture Research Center (ISTAR), Instituto Universitário de Lisboa (ISCTE-IUL), 1649-026 Lisbon, Portugal; 2 Inov Inesc Inovação—Instituto de Novas Tecnologias, 1000-029 Lisbon, Portugal

**Keywords:** information sharing, artificial intelligence, blockchain, medical registry, security, smart contract, encryption

## Abstract

Data sharing in the health sector represents a big problem due to privacy and security issues. Health data have tremendous value for organisations and criminals. The European Commission has classified health data as a unique resource owing to their ability to enable both retrospective and prospective research at a low cost. Similarly, the Organisation for Economic Co-operation and Development (OECD) encourages member nations to create and implement health data governance systems that protect individual privacy while allowing data sharing. This paper proposes adopting a blockchain framework to enable the transparent sharing of medical information among health entities in a secure environment. We develop a laboratory-based prototype using a design science research methodology (DSRM). This approach has its roots in the sciences of engineering and artificial intelligence, and its primary goal is to create relevant artefacts that add value to the fields in which they are used. We adopt a patient-centric approach, according to which a patient is the owner of their data and may allow hospitals and health professionals access to their data.

## 1. Introduction

Currently, there is a rising trend in the usage of mobile smart devices to store and manage users’ most sensitive data [1]. Nowadays, these intelligent gadgets gather, analyse, and store personal, financial, and even health-related information. In addition, the range of sensors available on smartphones, smartwatches, and smart bands has risen significantly. As such, user health data collection has reached a level never before seen—the standard gateway that combines all this data in the user’s smartphones (paired with other end-user devices) and a collection of different backend systems.

Blockchain can play an important role in remote health, certifying trusted devices, and storing and securing personal patient data [2]. This technology can be applied to an individual’s electronic health record (EHR), a compilation of health-related data that contains details about their personal (e.g., name, age, gender, weight, and billing information) and medical history, medications, and health problems (such as illnesses). One of the healthcare systems’ main issues is preserving medical data confidentiality and privacy, both at rest and in motion [3]. These systems need to share medical data securely because such data are frequently sensitive (and especially attractive to cybercriminals) and needs to be protected against unauthorised access (e.g., without resulting in leakage of patient data). Making an index for EHRs and encrypting them before they are uploaded to a public or community cloud is a typical but naive way to share medical data. The disadvantage of this strategy is that various data suppliers create indices differently, preventing data sharing across various medical organisations and individuals. Furthermore, the cloud provider might not be completely secure and may be exposed to attacks. A workable solution based on blockchain and distributed ledger technologies (B&DLT) may contribute to creating and using structured contracts for data access, standardised audits, and cryptographic algorithms to maintain data security and integrity.

Patient privacy may be jeopardised if data from EHRs leaks (e.g., medical conditions). B&DLT has the potential to be utilised to facilitate the secure and reliable sharing of such data because, in general, the majority of EHR data remain unmodified once they are posted to the system. As a result, strongly protected EHRs saved on the B&DLT can be accessed with higher reliability by many collaborating medical institutions and individuals (such as doctors, hospitals, labs, and insurance companies).

The increase in sub-specialisation in healthcare has led to the diversifying of patient care between different institutions and geographical areas. While primary care and palliative care are predominantly delivered within the scope of residence, secondary and tertiary care are provided by specialised institutions without any geographical or institutional relationship. In addition, the existence of different legal and administrative statutes, i.e., state-owned, charity, and private, among the healthcare providers further hampers communication and access by the other stakeholders.

Secondary and tertiary care medicine is based on upending technology that is only available in a restricted number of institutions. Furthermore, the progressive sub-specialisation of medical care has made it difficult, if not impossible, to deploy the necessary medical specialists and technologies in the same institution. Given the high financial costs of high-end technology, patients, doctors, and health professionals must move between institutions to match patient needs, human resource skills, and equipment availability. 

Additionally, the smartphone serves as a general health data gathering, aggregation, and storage device in this user-centric privacy environment and may be coupled with additional devices [4]. In the proposed system, the smartphone will collect and securely store all of the user’s sensitive health data in an encrypted and secure data vault (using multi-factor unlocking mechanisms). It will act as a personal generic data gateway between data processors (particularly large public hospitals of the national health system) and the user-anonymised data. This is one of the major contributions of our work.

We apply our approach to a Portuguese hospital (Hospital de Santa Maria) and check the data exchange between this hospital and private clinics. Data from patient records may be utilised at three levels to generate a personal knowledge graph in AI self-learning systems. The first (pre-existing, context-based) portrays commonplace occurrences, activities, states, and objects. The second (dynamically changed semantic modelling-based) is in charge of modelling semantic information to facilitate customisation. The third (dynamically changed feature-based) reflects low-level features and is mainly used for classification purposes. The extracted patterns will be correlated with data from other sensors and with specific situations, habits, and emotional states to improve the system’s overall performance via a personalised learning procedure that will learn the user’s baseline signals and adjust thresholds for signal abnormalities and decaying rhythms, thereby grading health risks.

This approach can be distinguished from other approaches, such as those presented in the review [5], as a system developed with identity management, coarse-grained, with data authentication and encryption, using consortium as the blockchain type and smart contracts, while storing data on the chain, and with interdomain interoperability. The system aims at secure and private interoperability between health data stakeholders, showing a Proof-of-Concept (PoC) among different health entities regarding health information sharing, not being the supply chain, as presented in the study [6]. All of these solutions were developed on Ethereum BCT since, in the literature, it is stated to be the best in terms of documentation, support, development, and scalability [7], and is one of the most used in healthcare applications [8], being flexible in terms of data storage and enabling different types of data to be stored via any smart contract [9].

## 2. Literature Review

This state of the art was produced by following the PRISMA (preferred reporting items for systematic reviews and meta-analysis) methodology [10], and with the research question “What is the state of the art on Data sharing using blockchain technology in Healthcare?”.

We conducted a search process on Scopus and the Web of Science Core Collection (WoSCC), and through June of 2022 all the results had to be articles published between 2012 and 2022 and written in English. 

The search strategy was based on one query made with different research focuses. This method allowed us to observe the number of articles in both databases, considering the concept, context, and population under study. 

For this review, only review articles were considered. Therefore, grey literature, conference papers, workshops, books, and editorials were excluded, as well as works unrelated to the topic.

The data were managed and stored using Zotero and Microsoft Excel. These data were title, author, year, journal, subject area, keywords, and abstract. A qualitative assessment was conducted based on the above results for data synthesis and analysis. All the databases—Scopus and WoS—were searched systematically for published work related to the concept of “Blockchain”, with the target population “Health Care” and within a “Data Sharing” study context.

The research was carried out by searching the existing literature regarding the concept, target population, and context of this study in Scopus and the Web of Science, as detailed in Table 1. The query was made in the individual databases and with the same restrictions and filters (it is important to note that the values corresponding to the queries still include duplicate articles).

From this, we can see that when the query is made using the keywords from each column (Concept AND Population AND Context AND Limitations), 124 documents are returned.

After performing a manual process to identify significant subjects by their research questions, identifying the outcomes and removing the duplicates, 22 documents were obtained. Our research systematisation considered year, area, RQ topic, and a short description.

**Figure 1:**
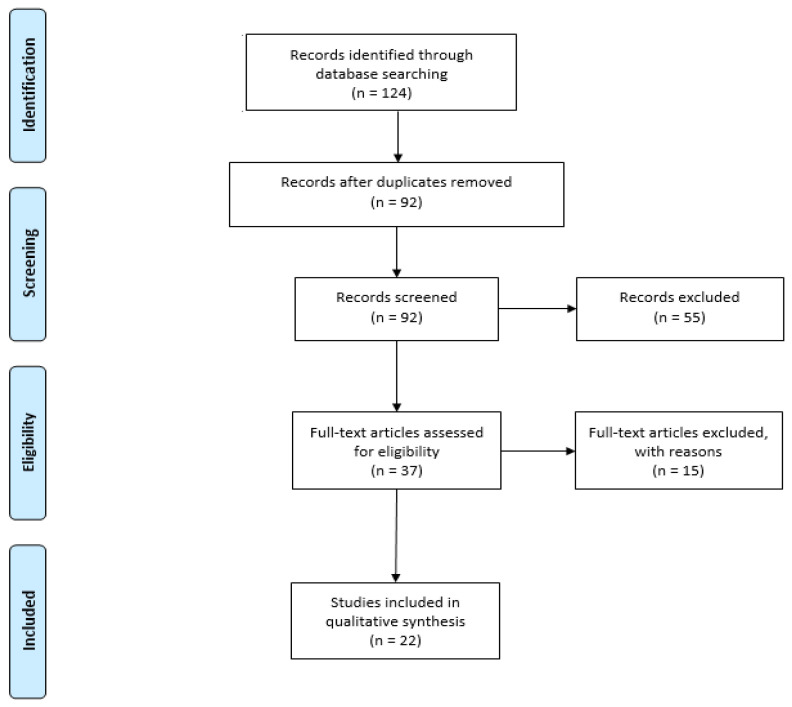
PRISMA process used in the literature review process.

Figure 1 shows the PRISMA workflow diagram from the total number of articles studied.

**Figure 2:**
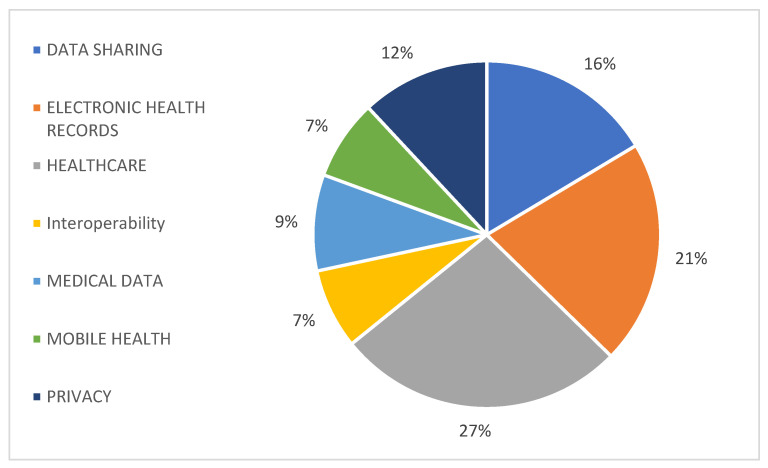
Publications identified based on main topics.

Because the goal of this article is to identify the use of blockchain technology in data sharing in healthcare, a list of the main topics discussed in each of the reviewed articles is described in Figure 2, where the focus on the sharing of healthcare and medical data can be observed.

### Goals and Outcome Analysis

**Reviews**—The reviews [11,12,13] assume that blockchain technology can be used to assess the constraints related to information integrity in the short and medium term. By safeguarding the data on the ledger, blockchain technology can cut down on loss and avoid data falsification [14], demonstrating that it is an efficient technology [15]. However, it does not ensure the information’s credibility in the first place and would have a number of drawbacks as a long-term fix for keeping reliable digital records. There is a need for further research [16] on these topics.

Examining and classifying the advantages and risks of using blockchain technology in the healthcare sector, studies [5,17,18] concluded that blockchain technology might promote patient-centric control of healthcare data sharing over institution-centric control. The researchers looked at how blockchain technology transforms the healthcare industry by providing digital access rights, patient identity throughout the network, and data immutability, and by processing a sizable amount of medical data. Bigini et al. [19] discuss the difficulties in achieving user-centricity for these integrated systems, indicating potential future courses for complete user ownership of data. The authors conclude that blockchain can be the technology that drives the development of long-lasting and independent platforms for data exchange that can respect privacy and contribute to the achievement of goals such as user-centricity, security, scalability, and interoperability. Review [20] found that blockchain could better match cloud-based health record management while preserving data security and privacy.

Following a survey of the literature on healthcare management systems, Wu and Tsai [21] suggested two techniques for network security. They also recommended creating rules for healthcare data and utilising a distributed system to handle them.

Dubovitskaya et al. [22] examined the drivers, benefits, and constraints, as well as the obstacles and upcoming difficulties encountered while using cutting-edge distributed ledger technology. Blockchain can potentially improve data [6] and EHR management [23] as well as data exchange (for medical research and primary care), and can optimise the pharmaceutical supply chain by giving the applications traits such as transparency, traceability, and immutability. Blockchain, however, cannot ensure the security and privacy of any data [12]. As a result, it is never suggested as a stand-alone technology, but rather as a system that uses cryptographic techniques.

Blockchain is a practical technology that may enhance healthcare data sharing and storage systems. However, many healthcare organisations are still unwilling to implement the technology due to the attendant risks, including security and authorisation concerns, interoperability problems, and a lack of technical expertise in blockchain technology, despite the fact that it may improve company processes, enhance patient results, reducing expenses, and standardise the entire procedure [24].

**Implemented Systems**—Regarding the systems found in the literature that have been implemented using the Hyperledger Fabric blockchain technology, Daisuke et al. [25] worked on medical records, transmitting medical data to the Hyperledger blockchain network. These medical records had been gathered using smartphones. In their approach, they sought to guarantee that medical data were recorded on the blockchain. MedChain [26] uses peer-to-peer networks and blockchain to share medical data. 

Jamil and colleagues discussed drug regulation problems and how to standardise medications and conduct drug record transactions on a blockchain. They recognised the challenges of identifying fake medications in their study and suggested using blockchain to identify fakes [27].

With blockchain technology and a microscope sensor, Lee and Yang developed a management system for fingernail examination. Over the developed system, they implemented blockchain technology that allowed the confidentiality and privacy of user data and the tracking and recording of system changes through a ledger [28].

In a presentation on consent management in e-health contexts, Philippe et al. [29] suggested blockchain as the safest and most dependable way to handle medical data. 

## 3. Patient-Oriented Health Data Management and Interoperability

**Figure 3:**
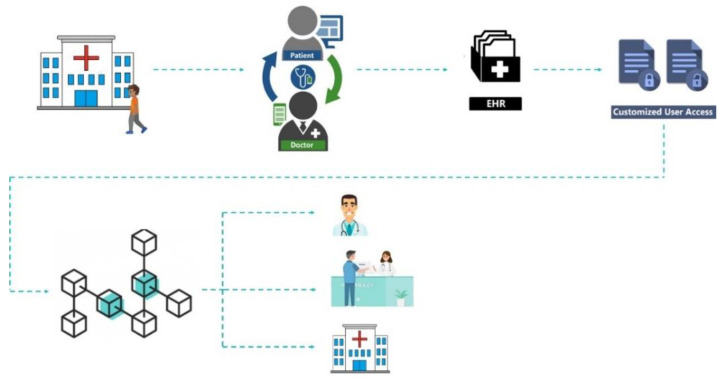
Information flow where patient data are stored on a private blockchain and patients allow access to their data.

In this article, we present a purpose-built solution based on hospital and patient privacy and security needs that leverages a combination of cryptographic technology to enable user and group-based secret sharing. Each datum in our blockchain system has a single user (owner) who can share that data with other users or groups at varying levels of access (summary versus complete data). Each datum consists of a description, which is viewable by anybody on the blockchain network, a summary, and content, and is stratified at different access levels to limit full access and only allow summary visualisation. As a result, summary access only offers the receiver a view of the descriptor and summary, whereas full access gives them visualisation of all three components. Individuals can share data with other users and groups and accept requests from other users at any access level modelled by a system. When a user grants data access in response to a request, a cryptographic artefact is exposed to the receiver so that only that receiver can view data at the stated level of access. Our system ensures that sensitive data, such as private and condidential documents, are never exposed on the B&DLT, which is necessary to maintain the privacy and security of user-controlled data. As an added security measure, our system preserves the fundamental property of revocation, which allows the data owner to revoke access to a piece of shared data, with the assurance that even a receiver’s private key combined with the raw blockchain transaction data will not be enough to gain access to the data. Because most blockchain implementations replicate the entire transaction ledger onto each node, the potential attack surface multiplies by the number of nodes in the network. A robust encryption scheme as part of a blockchain-based data-sharing system is essential from a security standpoint. Though our current system implements document-level access controls, we built the underlying architecture to facilitate attribute-based sharing. Figure 3 displays the process of sharing information through the blockchain, highlighting the ways in which it differs from more traditional solutions. Blockchain allows decentralisation and data privacy in a trustworthy and transparent environment through encryption and control mechanisms. The blockchain safeguards transparency by storing information that cannot be altered without recording the changes made. Data sharing among different entities should be carried out in a patient-centred manner because the patient owns his health records and can allow others to access his information. Data traceability is also available using this approach because each transaction is recorded in a chain of blocks. This method would need to capture the structure of submitted documents (patient information) in the underlying smart contracts in a way in which not all data fields are equally treated and sensitive areas are independently processed from the rest of the document.

Patients, physicians, and health professionals can navigate to different institutions and geographic areas, and the data remain in each institution, eventually, with access to patients or physicians outside. Figure 3 shows the proposed information flow, where patient data go through a chain of blocks and patient-related data access is granted to different health institutions. For example, patient and doctor appointments generate EHRs that can be associated with others in the blockchain. The patient later releases this information to other medical institutions or other doctors.

Patient records in healthcare institutions have multiple layers of information concerning the different stakeholders involved, and with diverse needs relating to confidentiality, data protection, ethical concerns that are typified in professional ethical principles, deontological rules for doctors and health professionals, institutional standard operational procedures, and national and European by-laws.

Patient records include personal information shared with the physician and healthcare provider based on confidentiality as well as information related to the working relationships among physicians or other healthcare providers, administrative data, procedures, financial and commercial data co-payments, and costs of employed material.

Patient records are relevant for the physician’s diagnostic hypothesis, therapeutic options, and healthcare plan. Patient records also retain laboratory data and procedure records, such as surgical or anaesthetic protocols. Patient records on specific procedures and exams, such as imaging or pathology, include multiple layers of information: (1) raw data obtained using the equipment; (2) images displayed by the equipment; (3) selected images for the report; and (4) the medical report itself. These different layers are available in entirely different formats and protocols, including vendor-specific image software, medical image software in DICOM format, and image analysis software, with which still or cine images may be viewed, and this is further complicated by the medical report, which is in text format. All these data are stored on the blockchain, and the patient controls the data access.

Data extraction and communication must include decisions concerning the relevant and appropriate information to extract, combine the multiple formats and presentations, and respect the proper ethics, data protection rules and regulation, and by-laws.

Most people have several health events and may visit multiple public or private healthcare facilities, namely hospitals, primary care units, or laboratories. Keeping track of all events and registered information can be a challenge. However, a personal health record can gather, store, track, and manage all that information in one easily accessible location.

A personal health record is simply a collection of health information about an individual. Some patients keep records of medical papers, such as prescriptions, X-rays, immunisation records, or lab results. These records can be considered essential personal health records, but patients rarely have them when needed.

Storing the EHR on a secure platform such as a blockchain can help overcome this problem by making the information accessible anytime via web portals or smartphone apps. The patient controls access to the information and may be able to manage, track, and participate in their healthcare.

An EHR’s main focus is to enable individuals to manage their health information and manage who can access this information. This accessibility of health information in an EHR may facilitate appropriate and improved treatment for conditions or emergencies away from an individual’s usual healthcare provider. For example, it can be a lifesaver in an emergency. Patients can quickly give first responders vital information, such as diseases for which they are being treated, medications they take, drug allergies, and contact information for the responsible doctor.

The personal health record (PHR), a collection of EHR data, includes information on all health service visits and personal information collected by wearable devices or sensors. For example, concerning health services, hospital units, primary healthcare units, rehabilitation centres, laboratories, or imaging centres, every time a patient has contact with one unit, administrative and clinical information is registered in the provider repositories. After the discharge or end of the connection, the patient can activate the integration of the recorded data into his PHR repository through the PHR web portal or app, being by default only available for him. Patients can also have personal health data gathered from multiple devices such as wearables, smartphones, or sensors, and this personal data can also be uploaded to their PHR. It is essential to point out that all data pulled to the PHR are exclusively owned by the patient by default.

All data stored in a PHR benefit patients, enabling them to track and manage their health history. Nevertheless, it is also of enormous value for other stakeholders, such as health organisations, insurance companies, governmental entities, and even research institutions. Once again, in the PHR portal or app, patients can control who can access their information and which level of information can be accessed. There is the possibility of different types of access that depend on the kind of organisation and the purpose of the entry (e.g., clinical, research, and commercial). For example, patients can activate the research share to grant access to their data for research purposes only, which means that any organisation registered as a clinical or academic research organisation will have access to this patient data. On the other hand, if a patient has a healthcare contact in a different institution that he usually does not attend or wants the share his data with his doctor, he can activate the clinical share so that his data can be shared for clinical purposes.

Because today’s world revolves around data, all available electronic information has a substantial commercial market. This is also true of clinical data; organisations seek information to develop clinical trials or study the impact of different insurance models. In these cases, patients, through their PHR, can sell pieces of their data for commercial purposes.

With all three types of access, patients can select the level of information to be accessed so that it can unlock different dimensions of their PHR for different purposes.

As referenced earlier, patients can pull their clinical data from the private or public institution’s repository to the PHR. For this integration to be possible, we assume there must be a technological interoperability layer that will allow data migration between different repositories. This interoperability layer will work by using different data connectors installed over the information system (IS), mapping the IS repository’s Internet Protocol (IP) address with the centralised PHR repository’s IP address, and establishing the connection between the two databases. This is a connection from the existing database to the blockchain register and vice-versa. This process needs customisation because there is no standardisation among medical databases. This connection must be granted at the level of the IS, so the integration focus is on migratable data, that is, data that the organisation must share with the patients. For a better understanding, let us look at a concrete example. Let us consider a public hospital that wants to permit patients to have their data in the PHR. This hospital can have multiple IS, administrative, clinical, and departmental systems. In this case, if the intention is to open all data to PHR integration, then the connector must be installed and configured in each IS so that, for each database, the integration with the central repository can be configured appropriately, considering the level of information allowed for integration and the context for each patient.

**Figure 4:**
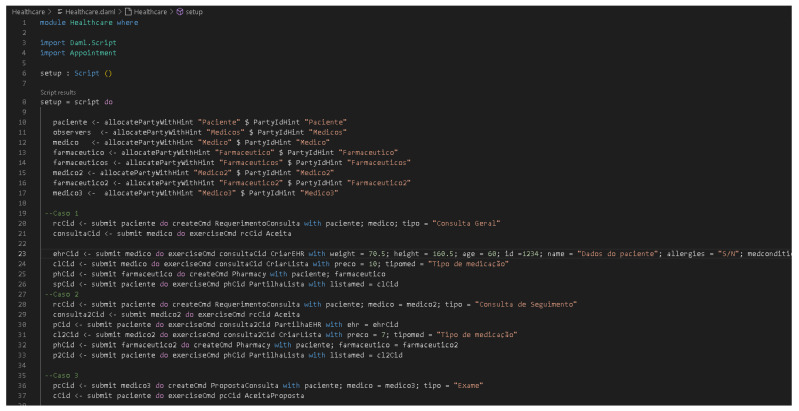
DAML code of the smart contract.

This distributed security architecture secures patient medical data confidentiality, integrity, and privacy. The current work proposes advancing the state of the art in user-related data protection by increasing the transparency of the developed system (by utilising open-source software solutions that enable third-party auditing) and implementing user-centric privacy solutions for end-user personal data access and usage control. In addition, it uses blockchain and distributed ledger technology to track user data and use permissions using rights expression languages and smart contracts. Each entity’s functions are as follows:

- **Patient**—A patient is also a data user. For example, consider a patient who registers with a hospital to visit a doctor. The hospital’s server k (Server[k]) produces and returns to the patient a certificate i (Cert[i]). Simultaneously, the server puts I = H(i) (H is the hash function using i) in the doctor j’s service registration list. Here, analogous to a license plate, it allows the doctor to produce medical records for patient I (see Figure 4, where we show this implementation on DAML). When the patient visits the doctor, he will provide the doctor with a certificate authorising the doctor to create their personal medical information and access his historical medical data.
- **Doctor**—After obtaining the patient’s agreement, the doctor is responsible for creating medical information for the patient and extracting data from the patient’s medical information. Additionally, to ensure the security of interoperable data, the patient and doctor agree on an access policy for encrypting the medical information and a key for generating the ciphertext. The ciphertext comprises two parts: the ciphertext of the patient’s medical records, which are saved on the blockchain, and the ciphertext of the keyword, which is recorded on the smart contract.
- **The requester of data**—The data requester is the data user, e.g., a scientific research organisation, medical insurance firm, or member of the patient’s family, who may receive access to relevant records provided they comply with the applicable access policy.
- **A distributed ledger technology network**—Our study is built on a blockchain network that executes smart contracts in a distributed way without depending on central entities, which is necessary to ensure the safe storage and exchange of electronic medical information. We use the Ethereum blockchain to increase system efficiency, since it is the most used [7]. In our PoC, we implemented six nodes with data from three Portuguese hospitals (two public other private), a private clinic, a research centre, and a pharmacy. For the time being, we exclude miners from our plan.

**Figure 5:**
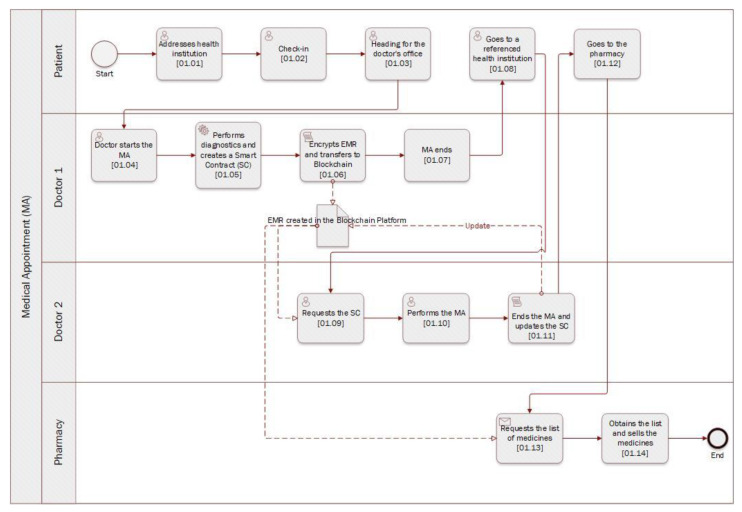
Information flow chart.

Let us check the following information flow of Figure 5: (1) The patient goes to the hospital for an appointment with the doctor who will record his data [01.01]; (2) The doctor creates a smart contract on the blockchain and stores a hash value of the signed medical data as well as a digital signature, used to confirm that the message came from the stated sender (its authenticity) and that it has not been changed [01.05]; (3) The doctor encrypts the medical record and uploads it to the blockchain according to the access policy agreed with the patient [01.06]; (4) The blockchain returns the location of the medical records to the doctor; (5) The physician creates the necessary records, incorporating the ciphertext into the transaction, and publishes it on the blockchain; (6) After the transaction is confirmed, the doctor notes its address; (7) The data requester submits a record access request to the physician, who authenticates the data requester’s identity and adds it to the list of approved smart contract users [01.09]; (8) The physician distributes the required features and produces an attribute key, which is subsequently delivered to the data requester; (9) The data requester generates a token and then places the smart contract as a parameter; (10) The smart contract verifies the identity of the data requester. If he is a valid user, the smart contract will provide him with the relevant search results; (11) The data requester parses the information about the blockchain transaction and searches for the file; (12) After obtaining the file location, the requester downloads the encrypted medical record from the blockchain; (13) The data requester determines whether its characteristics conform to the ciphertext access structure and, if so, decrypts and retrieves the medical record [01.11].

## 4. Implementation

To implement the scenario mentioned above, we concluded that using the Digital Asset Modelling Language (DAML) (http://www.daml.org/, accessed on 22 December 2022) language for smart contracts could effectively bring numerous advantages, e.g., the visualisation of all transactions performed on the blockchain to their conclusion. Therefore, we created a smart contracts architecture for accessing a patient’s medical data to be used by the various participants in the process, from hospital units to physicians and patients. This architecture contributes to the practical implementation of the required access control and interoperability architecture since most people have a series of health events and may visit several public or private health facilities, namely hospitals, primary care units, or laboratories. Keeping track of all the events and recorded information can be a challenge. However, with a personal health record, it is possible to gather, store, monitor, and manage all this information in one easily accessible place. Therefore, we can conclude that blockchain can facilitate the development of an RGPD-compliant EHR management system by encoding a set of rules into a smart contract that ensures that sensitive patient data cannot be shared or used without proper authorisations.

Figure 4 shows the DAML source-code (initial part) of the smart contract. We can check the roles of each stakeholder and then the three cases described and implemented. Based on the stakeholder’s role, the source-code for the implemented cases are mainly authorisation to access, register, and correct information in the patient EHR based on a pre-defined information flow. Thus, DAML allows the easy flow of information.

**Figure 6:**
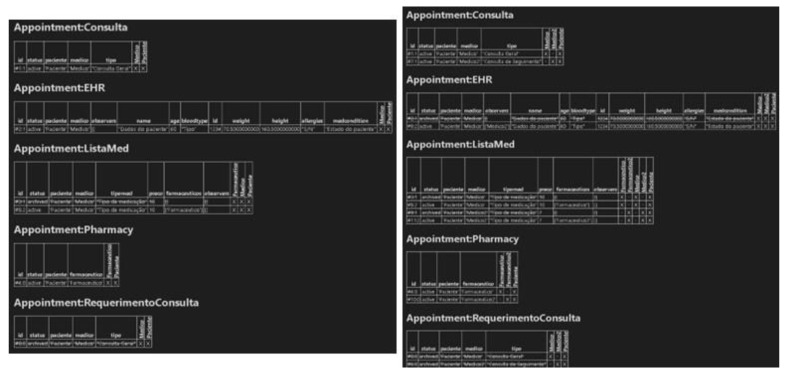
Application print screen for case 1 (left image) and case 2 (right image).

Considering a real scenario, first, the patient makes a request for an appointment, integrating the following parameters: “type”, “patient”, and “doctor”. The doctor then agrees to make the appointment, obtaining the “Appointment” contract and adding the “type” parameter. Thus, as the consultation occurs, the physician creates the “EHR”, which aggregates all of the patient’s biometric data (i.e., name, age, blood type, identification, weight, height, allergies, and medical conditions). After creating the EHR, the physician will also create a list of medications, called “ListaMed” in the contract, integrating the following parameters: “tipomed” (the type of medication) and “preco” (the price of the medication). Finally, it is necessary to share this list of medications with the pharmacist, so a pharmacy contract will be created. This contract integrates a pharmacist, where the patient will go to buy the medications prescribed by the doctor in that list of medications previously created (Figure 6, left screen application).

The patient makes an appointment request, in which the appointment will be a followed-up of the previous one and will be performed by a second doctor, called Physician2, and in which it will be possible to observe the contract “AppointmentRequest”. Next, the physician agrees to perform the consultation, thus originating the “Consultation” contract. However, at this stage, it will no longer be necessary to create a new “EHR”, but rather, the “EHR” previously created by the first doctor will be shared with the second doctor, the patient having agreed to make it available to the second doctor. The second doctor will then create a medication list with a different price and type of medication than the first doctor’s previous list. Consequently, following the previous logic, the “Pharmacy” contract is created, integrating a second pharmacist, called “pharmacist2”, in which the list of drugs created by the second doctor will be shared with this second pharmacist (Figure 6, right screen application).

**Figure 7:**
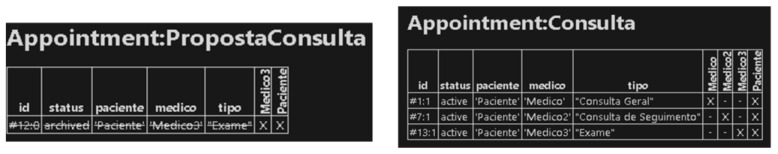
Application print screen.

Finally, a scenario is represented where, after all the consultations, a third doctor, called doctor3, proposes an exam to the patient, which the patient accepts and undergoes, thus ending the workflow process (Figure 7 ).

## 5. Discussion

It is well-known that patient data are maintained in various insecure forms across conventional healthcare delivery models, providers, labs, payers (i.e., insurance companies), and pharmaceutical firms, with no consistency or interoperability of record keeping. This has resulted in data breaches and in the current state of chaos in health information interchange. Inadequate infrastructure for data exchange has also stymied medication innovation and public health studies. Attempts to remedy this problem have generally imposed a new common standard throughout the ecosystem. These initiatives have failed because they were swiftly rejected by regulators and lobbyists, and suffered from patient disinterest. Due to the inadequate processing and interchange of health data, the tailoring of medical treatment to a patient’s characteristics, wishes, and expectations has not been widely adopted. Precision medicine (or customised medicine) has long been regarded as the future of healthcare. Business players have invested significant resources in developing individualised healthcare solutions, only to be hampered by the present system.

In this article, we reviewed the healthcare industry’s current demands and the present system’s inadequacies and offered Ethereum-based solutions for healthcare administration. We provided an overview of the status of personalised medicine, highlighting issues concerning the present healthcare system that impede the implementation of personalised medicine, and illustrating how our built approach addresses these concerns. As a result of these factors, specific healthcare departments, such as paediatrics and general surgery, have more significant expenditures than others.

## 6. Conclusions

Blockchain is a safe and dependable platform for secure data exchange in the financial sector, supply chain management, food industry, energy sector, Internet of Things, and healthcare. This article proposes a blockchain to allow the exchange of medical records in a secure and controlled process. Different medical workflows have been devised and executed utilising the Ethereum blockchain platform, and these have incorporated intricate procedures, including surgery and clinical studies. They also requires obtaining and maintaining a significant volume of medical data. Furthermore, within the execution of the workflows of the medical smart contract system for healthcare management, the related cost has been assessed for this system in terms of a feasibility study which has been extensively provided in this article. This endeavour will assist numerous active stakeholders within the medical field to offer better healthcare services and optimise costs.

Our smart contract-based healthcare management system has shown how decentralisation concepts may be utilised in the medical ecosystem to handle massive amounts of data and speed up complicated medical processes. We present a novel method for medical record management by using smart contracts to provide audibility, interoperability, and accessibility. Designed for maximum flexibility and granularity, this system permits the exchange of patient data and provides incentives for medical researchers to support the plan. We have presented possible blockchain uses for health data management. We developed a data management and sharing system based on medical needs. Using blockchain technology makes it possible to secure privacy, security, availability of EHR data, as well as fine-grained control of access. The ultimate objective of using blockchain in the manner described in this study is to enhance healthcare procedures and, therefore, patient outcomes. Blockchain technology may assist in various ways, including lowering transaction costs via smart contracts, which are embedded general-purpose protocols that streamline operations, minimise administrative burdens, and eliminate the need for intermediaries. Other blockchain initiatives seek to enhance the gathering, utilisation, and exchange of health data from patients, researchers, and subprocessors. Our proposed solution utilises blockchain technology to establish an iterative, scalable, safe, accessible, and decentralised healthcare ecosystem. This will let people quickly and securely share medical information with physicians, hospitals, research groups, and other stakeholders while preserving complete control over the privacy of their medical data. This would address several problems with the present healthcare system, including data, legacy network inconsistency, unstructured data-gathering challenges, unreasonably high administrative expenses, a lack of data security, and ignored privacy concerns.

## Figures and Tables

**Table 1:** Literature process outputs.

Concept	Population	Context	Limitations
Blockchain	“Health Care”	“Data Sharing”	“literature reviews”
			2012 to 2022
			journal papers only
290			
124			

## Data Availability

All the data can be used on request.
